# Lung cancer risk from radon in Ontario, Canada: how many lung cancers can we prevent?

**DOI:** 10.1007/s10552-013-0278-x

**Published:** 2013-08-28

**Authors:** Emily Peterson, Amira Aker, JinHee Kim, Ye Li, Kevin Brand, Ray Copes

**Affiliations:** 1 Public Health Ontario, 300-480 University Ave., Toronto, ON M5G 1V2 Canada; 2 Dalla Lana School of Public Health, University of Toronto, 155 College St., 6th Floor, Toronto, ON M5T 3M7 Canada; 3 Telfer School of Management, University of Ottawa, 55 Laurier Avenue East, Ottawa, ON K1N 6N5 Canada

**Keywords:** Radon, Lung cancer, Ontario, Canada, Burden of illness

## Abstract

**Purpose:**

To calculate the burden of lung cancer illness due to radon for all thirty-six health units in Ontario and determine the number of radon-attributable lung cancer deaths that could be prevented.

**Methods:**

We calculated the population attributable risk percent, excess life-time risk ratio, life-years lost, the number of lung cancer deaths due to radon, and the number of deaths that could be prevented if all homes above various cut-points were effectively reduced to background levels.

**Results:**

It is estimated that 13.6 % (95 % CI 11.0, 16.7) of lung cancer deaths in Ontario are attributable to radon, corresponding to 847 (95 % CI 686, 1,039) lung cancer deaths each year, approximately 84 % of these in ever-smokers. If all homes above 200 Bq/m^3^, the current Canadian guideline, were remediated to background levels, it is estimated that 91 lung cancer deaths could be prevented each year, 233 if remediation was performed at 100 Bq/m^3^. There was important variation across health units.

**Conclusions:**

Radon is an important contributor to lung cancer deaths in Ontario. A large portion of radon-attributable lung cancer deaths are from exposures below the current Canadian guideline, suggesting interventions that install effective radon-preventive measures into buildings at build may be a good alternative population prevention strategy to testing and remediation. For some health units, testing and remediation may also prevent a portion of radon-related lung cancer deaths. Regional attributable risk estimates can help with local public health resource allocation and decision making.

## Introduction

 Radon is a colorless, odorless, gaseous decay product of uranium found normally in soil. It can accumulate in enclosed spaces such as homes, schools, and workplaces. The short-lived daughters of radon release ionizing radiation during radioactive decay, and long-term exposures have been linked to lung cancer in humans through epidemiological studies [1]. Radon is the second leading cause of lung cancer after smoking [2]. Buildings with high radon levels can be remediated to reduce exposure to people living and working in them. Also, building codes can facilitate reduced radon entry into homes as well as the installation of other control measures [2].

The US National Academy of Sciences has developed a method for conducting burden of illness calculations due to radon, estimating that 10–14 % of lung cancer deaths in the USA are due to radon [3]. Similar methods have been applied in Canada, and the most recent estimate suggests that 16 % of lung cancer deaths in Canada are due to radon [4]. We performed calculations to estimate the burden of illness due to radon for each health unit in the Canadian province of Ontario using methods proposed by Brand et al. [5] that account for uncertainty in risk estimates.

Ontario, with a population of approximately 12.9 million people, has 36 health units, varying in geographical and population size, which are responsible for public health protection within the province. Budget and general guidelines on the programs offered by health units are assigned by the province, but decisions about program design, resource allocation and action are made by the local health unit based on its assessment of population needs. A recent review of the literature on the public health decision-making process suggests that a lack of local level research is a barrier to the use of evidence in public health [6]. When a public health concern can vary from one local region to the next, such as radon, providing regional/local level estimates of the burden of illness is important for the use of evidence in the allocation of resources and design of prevention initiatives.

## Methodology

**Fig. 1:**
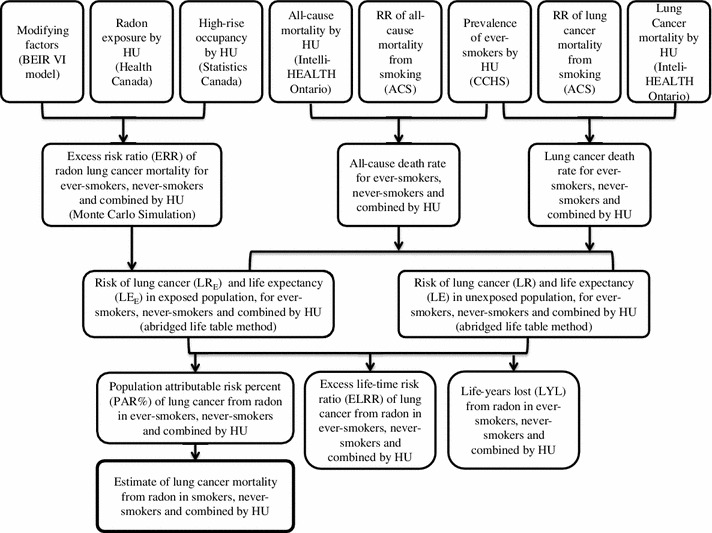
Flow diagram of methodology used to calculate the life-years lost (LYL), excess life-time risk ratio (ELRR), and population attributable risk percent (PAR %) of lung cancer deaths due to radon. *ACS* = American Cancer Society, *CCHS* = Canadian Community Health Survey

The method proposed by Brand et al. [5] was implemented using Ontario-specific data sources. These methods were based on the exposure-age-concentration model from the BEIR-VI report [3]. See Fig. 1 for an overview of the methods used to calculate the population attributable risk percent (PAR %), excess life-time risk ratio (ELRR), and life-years lost (LYL). Further analysis was performed to find the number of deaths attributable to radon exposure and the number of radon-attributable lung cancer deaths that can be prevented if all homes above 50, 100, 150 and 200 Bq/m^3^ were reduced to background levels. The methodologies and data sources are described below.

### Data sources

Radon exposure data were obtained from Health Canada’s Cross-Canada Survey of Radon Concentrations in Homes [7]. This survey was conducted in the fall and winter seasons of 2009/2010 and 2010/2011 using alpha track radon detectors with a minimum sampling period of 3 months. For this survey, there were 3,891 randomly selected samples taken across Ontario. This study used a health unit sampling frame, and participants were recruited via telephone. The overall study response rate was approximately 21 %. Readings below the detection limit of 15 Bq/m^3^ were assigned a random integer between 0 and 15 Bq/m^3^ with a mean of 7.5 Bq/m^3^ for the purposes of this study.

The percentage of people in each health unit living in apartment buildings (both below and above five stories) was calculated with 2006 Statistics Canada Census Tract (CT) data using ArcGIS. This was done by first obtaining the density of apartment dwellers for each CT then creating multiple intersecting polygons by overlaying CT boundaries with health unit boundaries and calculating the number of apartment dwellers in each polygon assuming uniform density within a CT. Finally, the polygons were reaggregated into health units and percentages for each health unit were obtained by dividing the total number of people in apartment buildings for each health unit by the health unit population.

The proportion of ever-smokers by health unit was calculated from the Canadian Community Health Survey [8] for 5-year age intervals using a derived variable indicating smoker type based on stated smoking habits using Ontario 2005, 2007, 2008 and 2009 data: the 4 years of data were pooled to enhance stability in the age-stratified estimates. Ever-smokers were classified as those who were daily, occasional, always occasional, former daily, and former occasional smokers. Never-smokers were defined as those who had never smoked.

Updated relative risk (RR) data for lung cancer mortality and for all-cause mortality due to smoking for both sexes combined were obtained from the American Cancer Society (M. Thun, personal communication). To calculate the pooled age-specific RRs, they used data from multiple cohort studies between 2000 and 2010. Because the updated RRs were only available for the age intervals of 55–59 and above, the RR for the interval of 55–59 was applied to the age intervals 45–49 and 50–54. All-cause and lung cancer mortality data for 2007 by health unit were obtained from intelliHEALTH Ontario.

### Methods

The equations we used are detailed in Brand et al. [5]. Using the exposure-age-concentration BEIR-VI model, the excess risk ratio (ERR) of lung cancer mortality for 5 year age intervals was calculated with health unit-specific radon exposure information and the percentage of the population living in high-rise buildings. A Monte Carlo simulation was used to estimate the uncertainty in ERR, and the number of iterations were modified slightly from the Brand et al. method to 150 × 900 × 900, in order to capture hyperparameter uncertainty, exposure uncertainty and inter-individual variability of the ERRs, yielding over 120 million simulated ERRs.

Abridged life-table calculations with 5-year age intervals were performed to find the lifetime risk (LR) and the life expectancy (LE) of lung cancer separately for ever-smokers, never-smokers, and combined (ever- and never-smokers) for each health unit. This was done using age-stratified health unit-specific lung cancer and all-cause mortality data, as well as the age-stratified proportion of ever-smokers in each health unit and the age-stratified RRs of lung cancer and all-cause mortality due to smoking. The previously simulated ERRs were then used in the life-table calculations to obtain the corresponding lifetime risk (LR_E_) and life expectancy (LE_E_) estimates of lung cancer in *exposed* individuals.

The ELRR and the PAR % of lung cancer from radon were calculated using the LR of lung cancer in the exposed and unexposed individuals, ELRR = LR_E_/LR-1, PAR % = (LR_E_ − LR)/LR_E_ × 100. Life-years lost (LYL) was calculated from the LE in the exposed and unexposed individuals, LYL = LE − LE_E_. This analysis was repeated for smokers, never-smokers, and combined for each health unit in Ontario. The estimated PAR %, ELRR, and LYL values for the province of Ontario were calculated by taking a population-weighted average of the health unit estimates.

To calculate the number of lung cancer deaths due to radon in both never- and ever-smokers combined, the mean combined PAR % was multiplied by the number of lung cancer deaths in that health unit in 2007. The mean number of lung cancer deaths due to radon for ever- and never-smokers separately were calculated by multiplying the number of lung cancer deaths in 2007 for each health unit with either 0.925 (ever-smokers) or 1–0.925 (never-smokers), and multiplying this number by the respective mean PAR % values, assuming that 92.5 % of lung cancers are in ever-smokers [3, 4].

In addition, all calculations above were repeated to determine the effect of remediating all homes above 100, 150 or 200 Bq/m^3^ to background levels. This was done by making all homes within each health unit at or above 100, 150 or 200 Bq/m^3^ similar to outdoor radon levels (by assigning them a random value between 10 and 30 Bq/m^3^). These action levels were chosen based on Canadian and international standards and the distribution of radon levels in health units across Ontario. For this analysis, 150 × 200 × 900 iterations were performed based on assessments of the stability in the initial PAR % analysis. In addition, we performed the same calculations for a hypothetical exposure scenario where all homes at or above 50 Bq/m^3^ were reduced to background levels to support calculations in relation to the effectiveness of building code interventions.

## Results

The measured radon levels from the Cross-Canada Survey of Radon Concentrations in Homes followed a lognormal distribution in Ontario with a provincial geometric mean of 43 Bq/m^3^ and a geometric standard deviation of 3.1 assuming all non-detects are assigned a value of 7.5 Bq/m^3^.

### Ontario

Table 1 shows the estimated PAR % of lung cancer deaths due to radon for ever-smokers, never-smokers, and combined, by quantiles and arithmetic mean for Ontario. Our estimated mean PAR % value for Ontario suggests that 13.6 % of lung cancer deaths within the province are attributable to radon. This estimate can be as low as 11.0 % (lower 95 % confidence limit) and as high as 16.7 % (upper 95 % confidence limit). As expected, our estimated PAR % values were higher in never-smokers with a mean of 21.9 % and lower in ever-smokers with a mean of 12.3 %.

**Table 1:** Estimated population attributable risk percent (PAR %) of lung cancer deaths due to radon, shown in quantiles and arithmetic mean, summarizing the uncertainty distribution, by smoking status; approximate mean number of lung cancer deaths attributable to radon in 2007; and the mean number and percentage of radon-attributable lung cancer deaths that could be prevented if all homes above stated radon concentrations were remediated to background levels

Geographical region	Smoking status	Population attributable risk percent (PAR %)						Lung cancer deaths attributable to radon (95 % CI)	Number (percentage) of radon-attributable lung cancer deaths that can be prevented			
		Quantiles							50 (Bq/m^3^)	100 (Bq/m^3^)	150 (Bq/m^3^)	200 (Bq/m^3^)
		2.5 %	5 %	50 %	95 %	97.5 %	Mean					
Ontario	Combined	11.0	11.4	13.5	16.2	16.7	13.6	847 (686, 1,039)	389 (46 %)	233 (28 %)	149 (18 %)	91 (11 %)
	Never	18.1	18.6	21.8	25.8	26.5	21.9					
	Ever	9.9	10.2	12.2	14.6	15.1	12.3					
HU1^ǂ^	Combined	21.7	22.2	25.2	29.0	29.6	25.3	21 (18.2, 24.9)	12 (57 %)	9 (42 %)	7 (31 %)	5 (23 %)
	Never	36.0	36.7	40.8	45.7	46.6	40.9					
	Ever	19.5	20.0	22.7	26.2	26.9	22.9					
HU2^ǂ^	Combined	6.9	7.2	9.0	11.2	11.6	9.1	24 (18.8, 31.3)	5 (21 %)	1 (4 %)	0 (0 %)	0 (0 %)
	Never	12.4	12.8	15.7	19.3	19.9	15.8					
	Ever	6.3	6.6	8.2	10.2	10.6	8.2					

Data are shown for Ontario and two selected health units with the highest (HU1) and lowest (HU2) PAR % values among all 36 health units. The number of deaths are rounded to the nearest whole number

Background levels were assigned a random value from 10–30 Bq/m^3^. ^ǂ^ We will make health unit identifiers available upon request

In 2007, there were 6,225 lung cancer deaths in Ontario, and we estimate that over 840 (= 0.136 × 6,225) of them were attributable to radon (95 % CI 686, 1,039). Our estimates show that over 700 (= 6,225 × 0.925 × 0.123) (approximately 84 %) of these radon-attributable lung cancer deaths were in the ever-smoker population.

The PAR % determines the theoretical percent of radon-attributable lung cancer deaths that could be prevented if all radon exposures were completely eliminated; however, this is not achievable in practice. Table 1 shows the estimated number of radon-related lung cancer deaths that could be prevented in Ontario if all homes above selected radon concentrations were effectively remediated to outdoor levels. It is estimated that 91 radon-related lung cancer deaths could be prevented if all homes in Ontario at or above 200 Bq/m^3^, the current Canadian guideline, were remediated to background levels. If this was done for all homes above 150 Bq/m^3^, an additional 58 radon-attributable lung cancer deaths could be prevented, and if all homes above 100 Bq/m^3^ were remediated as recommended by WHO [2], a further 84 radon-attributable lung cancer deaths could be prevented. If all homes in the province above 50 Bq/m^3^ were reduced to background levels, we estimate that 389 radon-attributable lung cancer deaths could be prevented, accounting for 46 % of the total estimated number of radon-attributable lung cancer deaths.

The distributions of the estimated ELRRs for ever-smokers, never-smokers, and combined for the general population of Ontario are shown in Table 2 in quantiles and arithmetic mean. The mean ELRR for both never- and ever-smokers combined in Ontario is 0.161, suggesting a 16 % greater risk of lung cancer at current levels of exposure in comparison with background levels. In the never-smoking population, the ELRR can be as high as 1.304 (97.5 % quantile).

**Table 2:** Estimated excess life-time risk ratio (ELRR) and estimated life-years lost (LYL) for Ontario shown as quantiles and arithmetic means, summarizing the uncertainty distribution, by smoking status

Smoking status	Excess life-time risk ratio (ELRR)						Life-years lost (LYL)					
	Quantiles						Quantiles					
	2.5 %	5 %	50 %	95 %	97.5 %	Mean	2.5 %	5 %	50 %	95 %	97.5 %	Mean
Combined	0.002	0.004	0.094	0.531	0.718	0.161	0.002	0.004	0.097	0.536	0.726	0.164
Never	0.003	0.006	0.169	0.960	1.304	0.291	0.001	0.002	0.039	0.217	0.294	0.066
Ever	0.001	0.003	0.084	0.470	0.635	0.143	0.002	0.004	0.116	0.650	0.879	0.198

The distributions of the difference in life expectancy between an unexposed and exposed population in Ontario, shown as LYL, are also displayed in Table 2 in quantiles and arithmetic means for never-smokers, ever-smokers, and combined. The average impact for the overall Ontario population is one-sixth of a life year lost and one-fifth of a life year lost in the ever-smoker population. The LYL estimates represent the *average* number of LYL due to radon, averaged across all people living in Ontario, including those who are not affected by radon.

### Health units

**Fig. 2:**
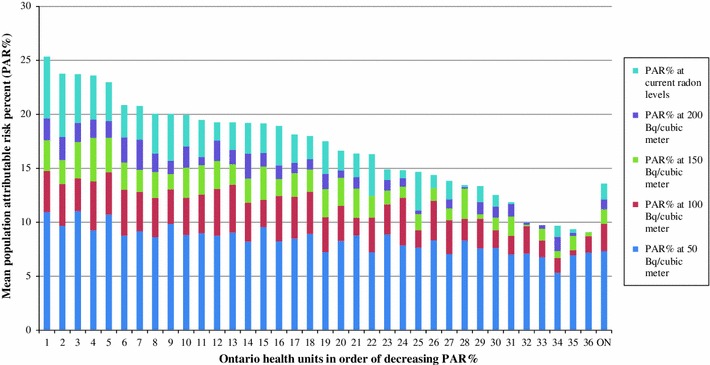
Mean population attributable risk percent (PAR %) of lung cancer deaths due to radon for both ever-smokers and never-smokers combined at current radon levels and various radon cutoffs by Ontario health units in order of decreasing PAR %. We will make health unit identifiers available upon request

There is a wide range in estimated PAR % values for health units across Ontario (Fig. 2). The results for two of the thirty-six health units with the highest (HU1) and lowest (HU2) estimated PAR % values are displayed in Table 1. The lowest mean estimated PAR % for ever-smokers and never-smokers combined is 9.1 %, while the highest is 25.3 %. However, in this case, population size differences between the two regions compensated for the PAR % differences resulting in similar lung cancer deaths attributable to radon between these health units, at 24 and 21 lung cancer deaths per year respectively. Although not shown in this example, variation in the number of radon-attributable lung cancer deaths between health units was observed with a mean of 25 and a standard deviation of 17.

In a remediation scenario where all homes above various radon levels are remediated to background levels, we see a large difference in the estimated number and percentage of radon-attributable lung cancer deaths that can be prevented, with a higher number in the health unit with the larger estimated PAR % (HU1) (Table 1). For example, if all homes in each health unit above 200 Bq/m^3^ were remediated to background levels, an estimated 23 % of radon-attributed lung cancer deaths could be prevented in HU1, while an estimated 0 % could be prevented in HU2. Regression analysis was performed to determine what factors explained the majority of the variation in PAR % between health units. As expected, measured radon concentrations drove most of this variation explaining over 70 % of it.

## Discussion

Our calculations suggest that a mean of 13.6 % (95 % CI 11.0, 16.7) or 847 (95 % CI 686, 1,039) lung cancer deaths in Ontario are due to radon, with approximately 84 % of these deaths occurring in ever-smokers. As expected, the estimated mean PAR % for Ontario is lower than the current Canadian estimate by Chen et al. of 16 % [4]. This is expected as radon levels are higher nationally than they are for Ontario [7], however, different methodologies were used for the two calculations. The 13.6 % mean PAR % estimate for this Ontario analysis is almost double the PAR % calculated for the Canadian population by Brand et al. [5], using the same methodology. The twofold difference appears traceable to the greater than twofold difference in average radon concentration assumed in the two analyses; data made available in 2012 have revealed higher average radon levels than were previously thought [7]. Our PAR % estimates for ever-smokers and never-smokers also appear consistent with these studies.

We predict that a public health strategy based on testing and remediation would theoretically only prevent 91 (11 %) radon-attributable lung cancer deaths each year if all homes above the current Canadian guideline of 200 Bq/m^3^ in Ontario were tested and effectively remediated to background levels. If the WHO guideline of 100 Bq/m^3^ was used, 233 (28 %) radon-attributable lung cancer deaths could be prevented annually. Wide variation between health units was observed in the estimated PAR % and in the number of radon-attributable lung cancer deaths that could be prevented through testing and remediation.

To our knowledge, this is the first study examining the burden of lung cancer from radon by health unit within a Canadian province that has the potential to inform public health action. The limitations of this analysis are common to other radon burden of illness calculations. The exposure data used for this analysis may not be representative of each health unit, given that for some health units, fewer than 100 samples were taken and radon levels can vary widely from home to home. The distribution of measured radon levels can impact both the burden of illness estimates and the theoretical number of radon-attributable lung cancer deaths that could be prevented with testing and remediation.

The analysis performed here also assumes a constant exposure to radon over one’s lifetime, i.e., that people reside in one home throughout their lifetime. If residential mobility was taken into account, the mean burden of illness estimates would not be impacted, but the results of the analysis would have less variability, with fewer estimates at the high and low ends of risk [9, 10]. By excluding mobility from the model, we may also be overestimating the number of radon-induced lung cancer deaths in health units with higher radon levels and underestimating the number in health units with lower radon levels, assuming people move between health units [9]. The use of the ever-smoker category, including current, occasional, and previous smokers, may be an oversimplification of the risk in this group, but we chose to use this category to remain consistent with the BEIR-VI report. The assumptions used for this model are discussed in the BEIR-VI report [3]. While these limitations are important, our model provides our best estimates of the burden of radon-associated lung cancer deaths in Ontario based on the most recent exposure data.

Our calculations suggest that the PAR % varies between health units and that testing and remediation may have a greater potential impact in some health units versus others. The combination of the PAR % results, number of radon-attributable lung cancer deaths and the estimated number of deaths that could be prevented through testing and remediation could assist in local level public health decision making and resource allocation. These data will be provided to each of the 36 health units. Given that the variation in PAR % estimates between health units are driven by the radon concentrations, health decision making and resource allocation could also incorporate radon concentration information in addition to the PAR % and estimated number of deaths. Based on their local results, health units may choose multiple options to address radon including but not exclusive of promoting testing and remediation, promoting building code changes, combining radon campaigns with smoking cessation campaigns, and a choice to focus limited resources on other public health issues.

Our results indicate that further health protection could be achieved with a lower indoor radon guideline; however, reliance on individual homeowners to perform testing, and then undertake remediation, is a significant disadvantage to this approach. One Quebec study estimated that only 6 % of homeowners would test for radon with a screening promotion approach [11]. Another study found that those who are at greater risk (i.e., smokers, unemployed people, and those living in homes with higher radon levels) are less likely to remediate their homes than others [12]. In view of this, we expect that our estimates of preventable radon-attributable lung cancer deaths at the various radon levels are optimistic. Despite this, testing and remediation is still an important message because it is the only applicable strategy for existing buildings. Other approaches to encourage testing and remediation may be more effective, such as pairing testing with real-estate transactions or providing financial incentives to homeowners[13–15], but there is little literature currently available to demonstrate how effective these options would be at reducing exposures. One of the issues to keep in mind with pairing radon testing with real estate transactions is that the timeline requirements for real estate transactions do not allow for longer term, minimum 3 month, testing that has been recommended by Health Canada to accurately assess exposure [16].

An alternative approach to promoting individual adherence to the radon guideline is to design and install effective radon-preventive measures into buildings during initial construction through mandatory building codes. Although this is a long-term approach, it is likely to be far more effective at the population level and more cost-effective than a retrofitting remediation approach [17, 18], and could drastically reduce the need for testing and remediation over time. Using Statistics Canada data [19], we estimate that within any given 5-year period, approximately 10 % of detached, semi-detached and row homes in Ontario are built new. Assuming that this growth rate (10 % over 5 years) remains constant, we estimate that if a new radon-related building code were implemented this year, in 37 years, 50 % of the homes in Ontario would be built to that standard. If at that point 50 % of the homes are indeed to standard, one can assume that 50 % of Ontarians would be exposed to ambient radon levels, while the remaining 50 % would be residing in houses representative of the old housing stock and thus arguably subject to the same average PAR % that we reported in Table 1 (for Ontario, combined)—namely 13.6 %. If we assume that those living in new (built to standard) homes are subject to levels at an ideal lower bound of 0 Bq/m^3^ and a higher bound of 50 Bq/m^3^, then we can calculate two population-weighted PAR %s that would apply as rough upper and lower estimates to the whole Ontario population in 2050: (0 × 0.5 + 1 × 0.5) × 13.6 = 6.8 % as the lower bound estimate and (0.54 × 0.5 + 1 × 0.5) × 13.6 = 10.5 % as the upper bound estimate using the results of our analysis of a cut-point of 50 Bq/m^3^. This suggests that if effective building codes were implemented, they could reduce between 23 and 50 % of the burden 37 years from now (keeping in mind an appropriate lag time between exposure reductions and a resulting reduction in burden, as is the case for all of our estimates). This strategy may also provide additional protection from other soil volatiles that contaminate indoor air. There are effective interventions for reducing radon entry into homes [20]; however, further research is needed to identify the most cost-effective and reliably efficacious interventions that can be installed at initial construction and are applicable to building conditions in Ontario [20, 21].

Some researchers have also argued that smoking cessation programs are a good approach to reduce the burden of illness from radon-related lung cancer deaths [22, 23]. Our calculations estimate that over 700 of the 847 radon-attributable lung cancer deaths in the province are among those who are ever-smokers. The combination of radon remediation and smoking cessation programs may also be explored as options to prevent radon-attributable lung cancer deaths in the province.

The advantages and disadvantages of each of these radon-risk-reduction strategies prompt careful consideration in determining the most appropriate approach for Ontario and each individual health unit. Despite the general paucity of environmental exposure data for the purposes of health planning and evaluation, in the case of radon, we have been able to provide an estimate of the risk of radon-induced lung cancer deaths to the Ontario population to support policy and future resource utilization. These burden of illness calculations can help prioritize radon initiatives across the province of Ontario.
